# Behavioral Health Risk Profiles of Undergraduate University Students in England, Wales, and Northern Ireland: A Cluster Analysis

**DOI:** 10.3389/fpubh.2018.00120

**Published:** 2018-05-07

**Authors:** Walid El Ansari, Derrick Ssewanyana, Christiane Stock

**Affiliations:** ^1^Department of Surgery, Hamad General Hospital, Hamad Medical Corporation, Doha, Qatar; ^2^Faculty of Applied Sciences, University of Gloucestershire, Gloucester, United Kingdom; ^3^College of Medicine, Qatar University, Doha, Qatar; ^4^School of Health and Education, University of Skövde, Skövde, Sweden; ^5^Utrecht Centre for Child and Adolescent Studies, Utrecht University, Utrecht, Netherlands; ^6^Unit for Health Promotion Research, Department of Public Health, University of Southern Denmark, Esbjerg, Denmark

**Keywords:** college students, gender, lifestyle, multiple behaviors, risk factors, cluster analysis

## Abstract

**Background:**

Limited research has explored clustering of lifestyle behavioral risk factors (BRFs) among university students. This study aimed to explore clustering of BRFs, composition of clusters, and the association of the clusters with self-rated health and perceived academic performance.

**Method:**

We assessed (BRFs), namely tobacco smoking, physical inactivity, alcohol consumption, illicit drug use, unhealthy nutrition, and inadequate sleep, using a self-administered general Student Health Survey among 3,706 undergraduates at seven UK universities.

**Results:**

A two-step cluster analysis generated: Cluster 1 (the high physically active and health conscious) with very high health awareness/consciousness, good nutrition, and physical activity (PA), and relatively low alcohol, tobacco, and other drug (ATOD) use. Cluster 2 (the abstinent) had very low ATOD use, high health awareness, good nutrition, and medium high PA. Cluster 3 (the moderately health conscious) included the highest regard for healthy eating, second highest fruit/vegetable consumption, and moderately high ATOD use. Cluster 4 (the risk taking) showed the highest ATOD use, were the least health conscious, least fruit consuming, and attached the least importance on eating healthy. Compared to the healthy cluster (Cluster 1), students in other clusters had lower self-rated health, and particularly, students in the risk taking cluster (Cluster 4) reported lower academic performance. These associations were stronger for men than for women. Of the four clusters, Cluster 4 had the youngest students.

**Conclusion:**

Our results suggested that prevention among university students should address multiple BRFs simultaneously, with particular focus on the younger students.

## Introduction

Major modifiable detrimental behavioral risk factors (BRFs) (e.g., tobacco use, unhealthy diets, physical inactivity, and harmful consumption of alcohol) are known to limit peoples’ capabilities. Nevertheless, to date, these modifiable BRFs still represent significant burdens among university students (1–5).

Studies of the health and wellbeing of university populations revealed different extents of clustering of lifestyle BRFs across students (3, 4, 6, 7). Among British university students, research has reported three distinctive health behavior risk profiles based on five lifestyle BRFs (4). Evidence indicates that single BRFs and especially alcohol consumption is related to poorer self-rated health and lower academic achievement of students (8). However, existing research that identified student clusters with specific health risk profiles, e.g., Ref. (3, 4) did not investigate the association between belonging to risky behavior cluster/s and poor health or lower academic outcomes. Such information would be relevant, as the propensity of the collective clustering of unhealthy behaviors exponentially exacerbates the risk for comorbidity in later life (8, 9).

Cluster analysis (CA) is a promising approach to assess students’ health-related lifestyle characteristics in a collective manner. CA is premised on shared characteristics to categorize a given population into mutually exclusive subgroups (clusters) for which the properties or patterns within a cluster are similar to each other than they are to properties within a different cluster (10, 11). Indeed, researchers have voiced that most health behavior research has adopted an approach where behaviors were studied in isolation and traditionally focused on individual risk behavior/s, in segregation from other BRFs (4, 7), despite that BRFs co-exist together and are related to one another (12). Limited research has explored clustering of lifestyle BRFs; and the studies that undertook such approach rarely focused on university students. In addition, few of such studies considered a wide/diverse range of BRFs; and rarely assessed the relationships between the emerging BRFs clusters and students’ self-rated health and academic performance.

To bridge these knowledge gaps and to add new insights to the limited research on clustering of BRFs, and its association with health and academic achievement of students, the current study employed a large sample of students at seven universities in three countries of the United Kingdom (England, Wales, and Northern Ireland) in order to: (1) identify and describe the clustering of five major lifestyle BRFs [health awareness, nutrition behavior, physical activity (PA), sleep, and alcohol, tobacco, and other drugs]; (2) characterize the student composition of each of the emerging clusters in terms of sociodemographics; and (3) examine the associations between the emerging BRFs clusters and students’ self-rated health and academic performance.

## Materials and Methods

### Ethics, Sample, and Data Collection

Ethical approval from the participating institutions (see below) was obtained prior to data collection. A self-administered general Student Health Survey collected health and well-being data through 2007–2008 (1, 3, 13, 14) during the last 10 min of the lectures. The research aims and objectives were explained in an information sheet delivered with the questionnaire to the participants. Students were informed that participation was voluntary and that by completing the questionnaire, they agreed to participate in the study. Hence, informed consent was obtained in accordance with the Declaration of Helsinki from all individual participants included in the study. Confidentiality was observed, participants were informed that the study was anonymous, no incentives were provided, and data were strictly protected at all stages. Representative sampling was achieved from each of the seven participating institutions, and an 80% response rate led to a sample of 3,706 undergraduate students (University of Chester *n* = 993, University of Gloucestershire *n* = 970, Bath Spa University *n* = 485, University of Ulster *n* = 475, Swansea University *n* = 406, Oxford Brookes University *n* = 208, and Plymouth University *n* = 169). Data quality assurance was optimized through centralized data entry using Teleform computer software.

### Measures

Similar to other general student health and wellbeing studies (3, 5, 13, 15), the data collection tool captured: sociodemographic information (age, gender, sufficiency of income, and type of accommodation); lifestyle features (PA, nutritional intake, restful sleep patterns, illicit drug use); self-rated health; and self-rated academic performance. The following questionnaire based on previous studies (3, 13) measured students’ health behavior and lifestyle, self-rated health, and self-rated academic performance, and are included in the CA of this study.

#### Health Awareness/Consciousness

Health awareness/consciousness was assessed by the question “To what extent do you keep an eye on your health?” with four response options (“Not at all,” “not much,” “to some extent,” and “very much”).

#### Nutritional Behavior

Consumption of fruits and vegetables was assessed with the question “How many servings of fruits and vegetables do you usually have per day (1 serving = 1 medium piece of fruit, 1/2 cup chopped, cooked, or canned fruits/vegetables, 3/4 cup fruit/vegetable juice, small bowl of salad greens, or 1/2 cup dried)?” with four response options (“I don’t eat fruits and vegetables,” “1–2,” “3–4,” and “5 or more” servings).

Consumption of sweets was measured by the question “How often do you eat sweets (chocolate, candy, etc.)?” with five response options (“Several times a day,” “daily,” “Several times a week,” “1–4 times a month,” and “never”).

The importance of healthy eating was measured with the item “How important is it for you to eat healthy?” with five response options from “Very important” to “Not at all important.”

#### Physical Activity

Three forms of PA (i.e., vigorous PA, moderate PA, and muscle strengthening PA) were assessed with the following questions: “On how many of the past 7 days did you: (1) participate in vigorous exercise for ≥20 min?; (2) participate in moderate exercise for ≥30 min?; (3) do exercises to strengthen or tone your muscles, such as push-ups, sit-ups, or weight lifting?” For each form of PA, students reported the number of days for which they engaged in any such activity (ranging from 0 to 7 days).

#### Sleep

Sleep/rest was assessed with the question “On how many of the past 7 days did you get enough sleep so that you felt rested when you woke up in the morning?” Students reported the number of days (ranging from 0 to 7 days).

#### Alcohol, Tobacco, and Other Drug (ATOD) Use

Alcohol (frequency) was assessed by the item “within the last 3 months, how often did you drink alcohol, e.g., beer?” with six response options “Several times/day,” “Everyday,” “Several times/week,” “Once a week,” “Less than once/week,” and “Never.”

Alcohol (binge drinking) was measured with the question “Think back over the last 2 weeks. How many times, if any, have you had five or more alcoholic drinks at a sitting?”

Alcohol problem drinking was assessed using the 4 standard items that form the CAGE screening test (16) for problem alcohol use with 2 response options (“Yes,” “No”). From the total score to these items, a binary variable was formulated, where a cut-off of scores ≥2 indicated presence of “Problem drinking,” while scores <2 indicated “No problem drinking” (16).

Smoking was measured with the item “Within the last 3 months, how often did you smoke (cigarettes, pipes, cigarillos, cigars)?” with response options “Daily,” “Occasionally,” and “Never.”

Illicit drug (ecstasy, marijuana, cocaine, heroin, crack, LSD, amphetamines) was assessed by the question “Have you ever use/used drugs?” with response options “Yes, regularly,” “Yes but only a few times,” “Never.”

#### Self-Rated Health

Self-rated health was assessed by asking “How would you describe your general health?” with five response options “Excellent,” “Very good,” “Good,” “Fair,” and “Poor.”

#### Self-Rated Academic Performance

Self-rated academic performance was measured by the item “How do you rate your performance in comparison with your fellow students?” There were five response options: “Much better,” “Better,” “The same,” “Worse,” and “Much worse.”

### Statistical Analyses

We undertook a two-step CA (11) based on 13 lifestyle BRFs (8 categorical, 5 continuous) using SPSS v23.0. Two-step CA combines pre-clustering and hierarchical methods to identify groupings that differ on criterion variables within a data set. This method is suitable for large datasets and can handle scale and ordinal data in the same model (16). In our clustering algorithm, we utilized a two-step procedure with a hierarchical clustering method, i.e., the Schwarz’s Bayesian Criterion to automatically determine the number of clusters. We employed two distance measures namely, log-likelihood (for categorical variables) and Euclidean (for continuous variables) (11). Within each cluster, we computed the percentage (%) for specific categories of lifestyle behavioral factors. Uniform categories were utilized across each cluster per behavioral factor to ensure accurate comparability of outcomes. For lifestyle behavioral variables in continuous data format, for example days per week of PA, the mean was presented for each cluster. We conducted Chi-square tests together with Cramer’s V test (17) to identify differences between the clusters in terms of sociodemographic characteristics and categorical BRFs. Analysis of variance (ANOVA) with *post hoc* pairwise comparisons using the Bonferroni method (18) was used to assess the significance of differences in BRFs that were in continuous variable format among the clusters. The ordinal regression (in statistical package STATA 14) examined the association between cluster membership (main exposure variable) and two dependent variables, namely: (1) students’ self-rated health and (2) students’ self-rated academic performance. Missing data in the original sample were handled through multiple imputation for non-response (19). We performed 20 imputations in SPSS v23.0 and utilized a complete sample of the twentieth imputation as basis for our analysis and results reported in this article.

## Results

### Health-Related Lifestyle Characteristics

Table 1 summarizes the sample’s gender aggregated lifestyle characteristics.

**Table 1:** Students’ health behavior and lifestyle characteristics by gender.

Variable	Whole sample	Women	Men	*p*

	*N*(%) or mean (SD)	*N*(%) or mean (SD)	*N*(%) or mean (SD)	
Health consciousness (*n* = 3,706)				<0.001
Very much	756 (20.4)	551 (19.1)	205 (24.9)	
To some extent	2,356 (63.6)	1,895 (65.7)	461 (56.0)	
Not much	559 (15.1)	420 (14.6)	139 (16.9)	
Not at all	35 (0.9)	17 (0.6)	18 (2.2)	

Importance of eating healthy (*n* = 3,706)				0.002
Very important	1,118 (30.2)	886 (30.7)	232 (28.2)	
2nd to very important	1,489 (40.2)	1,165 (40.4)	324 (39.4)	
3rd to very important	908 (24.5)	704 (24.4)	204 (24.8)	
4th to very important	161 (4.3)	111 (3.8)	50 (6.1)	
Not at all important	30 (0.8)	17 (0.6)	13 (1.6)	

Daily fruit/vegetable (*n* = 3,706)				<0.001
≥5	569 (15.4)	477 (16.5)	92 (11.2)	
3–4 servings	1,502 (40.5)	1,204 (41.8)	298 (36.2)	
1–2 servings	1,521 (41.0)	1,141 (39.6)	380 (46.2)	
I do not eat fruit and vegetables	114 (3.1)	61 (2.1)	53 (6.4)	

Consumption of sweets (*n* = 3,706)				<0.001
Never	77 (2.1)	47 (1.6)	30 (3.6)	
1–4 times a month	1,090 (29.4)	816 (28.3)	274 (33.3)	
Several times a week	1,523 (41.1)	1,206 (41.8)	317 (38.5)	
Daily	883 (22.5)	671 (23.3)	162 (19.7)	
Several times a day	183 (4.9)	143 (5.0)	40 (4.9)	

Physical activity (PA) (days per week)				
Vigorous PA for ≥20 min	1.9 (SD 1.8)	1.7 (SD 1.7)	2.4 (SD 1.9)	<0.001
Moderate PA for ≥30 min	2.1 (SD 1.9)	1.9 (SD 1.9)	2.5 (SD 1.9)	<0.001
Muscle strengthening/toning PA	1.8 (SD 2.3)	1.7 (SD 2.3)	2.1 (SD 2.2)	<0.001

Enough sleep/rest	2.8 (SD 2.1)	2.8 (SD 2.2)	2.8 (SD 2.0)	0.885

Substance/illicit drug use				
Smoking in past 3 months (*n* = 3,706)				0.007
Never	2,680 (72.3)	2,103 (72.9)	577 (70.1)	
Occasionally	442 (11.9)	318 (11.0)	124 (15.1)	
Daily	584 (15.8)	462 (16.0)	122 (14.8)	
Ever use/used drugs (*n* = 3,706)				<0.001
Never	2,580 (69.6)	2,130 (73.9)	450 (54.7)	
Yes, but only a few times	945 (25.5)	652 (22.6)	293 (35.6)	
Yes, regularly	181 (4.9)	101 (3.5)	80 (9.7)	

Alcohol				
Frequency of consumption, past 3 months (*n* = 3,706)			<0.001
Never	295 (8.0)	234 (8.1)		61 (7.4)
Less than once a week	843 (22.7)	730 (25.3)		113 (13.7)
Once a week	985 (26.6)	822 (28.5)		163 (19.8)
Several times a week	1,391 (37.5)	990 (34.3)		401 (48.7)
Every day	149 (4.0)	85 (2.9)		64 (7.8)
Several times a day	43 (1.2)	22 (0.8)		21 (2.5)
Binge drinking (≥5 alcoholic drinks at 1 sitting, last 2 weeks) (*n* = 3,706)				<0.001
No	3,227 (87.1)	2,578 (89.4)	649 (78.9)	
Yes	479 (12.9)	305 (10.6)	174 (21.1)	
Problem drinking (CAGE score) (*n* = 3,706)				<0.001
Non problem drinking	2,902 (78.3)	2,310 (80.1)	592 (71.9)	
Problem drinking	804 (21.7)	573 (19.9)	231 (28.1)	

### Clustering of Lifestyle BRFs Among Students

Cluster analysis generated 4 clusters (Tables 2 and 3). Clusters 1 and 2 were of almost even size (ratio of largest cluster to smallest = 1.1) and approximately twice the size of Clusters 3 and 4. As depicted in Table 2, the clusters differed significantly by gender. The percentages of female students were highest in Clusters 1 and 4 and lowest in Cluster 3. All gender differences between clusters were significant (χ^2^ tests, *p* < 0.001) except for the comparison between Clusters 1 and 3.

**Table 2:** Cluster properties by selected socio-demographic factors.

Variable	Cluster 1	Cluster 2	Cluster 3	Cluster 4

	The high physically active and health conscious (*n* = 1,070) *N*(%)	The abstinent (*n* = 1,201) *N*(%)	The moderately health conscious (*n* = 590) *N*(%)	The risk taking (*n* = 845) *N*(%)
Accommodation				*p* < 0.001
Alone	39 (30.2)	32 (24.8)	29 (22.5)	29 (22.5)
With partner	143 (31.8)	81 (18.0)	87 (19.4)	138 (30.7)
With parents	132 (23.9)	161 (29.1)	88 (15.9)	172 (31.1)
With roommates	228 (22.1)	234 (22.7)	331 (32.1)	239 (23.2)
Other accommodation	54 (29.8)	41 (22.7)	49 (27.1)	37 (20.4)
Disposable monthly income				*p* = 0.004
Always sufficient	57 (33.3)	32 (18.7)	37 (21.6)	45 (26.3)
Mostly sufficient	216 (25.5)	199 (23.5)	185 (21.9)	246 (29.1)
Mostly insufficient	188 (24.0)	191 (24.4)	204 (26.0)	201 (25.6)
Always insufficient	142 (26.3)	124 (23.0)	160 (29.7)	113 (21.0)
Gender				*p* < 0.001
Women	490 (27.0)	413 (22.8)	397 (21.9)	513 (28.3)
Men	122 (21.4)	136 (23.9)	203 (35.6)	109 (19.1)
Age				*p* < 0.001
Mean age (SD)	27.0 (SD 10.0)	24.1 (SD 7.7)	25.7 (SD 9.1)	22.6 (SD 6.4)

**Table 3:** Comparison of health behavior and lifestyle characteristics between four student clusters in the UK.

Health behavior/lifestyle variable	Cluster 1	Cluster 2	Cluster 3	Cluster 4

	The high physically active and health conscious (*n* = 1,070)	The abstinent (*n* = 1,201)	The moderately health conscious (*n* = 590)	The risk taking (*n* = 845)
Health awareness, very much or to some extent (%)	95.4^a^^,^^b^^,^^c^	89.3^e^	88.3^f^	58.8

Nutrition (%)				
Eating healthy ranked *very important, second*, or *third* in level of importance	99.5^a^	97.9^e^	100.0^b^^,^^g^	80.9^c^^,^^f^
≥5 daily fruit and vegetable servings	39.5^a^^,^^c^	6.6^e^	7.6^b^	2.6^f^
Moderate consumption of sweets (*never, 1–4 times a month, several times a week*)	79.1^a^	70.3^d^^,^^e^	96.1^b^^,^^f^	51.1^c^

PA, mean days per week (SD)				
Vigorous PA	2.6 (1.9)^a^	1.5 (1.5)	1.7 (1.6)^b^	1.5 (1.7)^c^
Moderate PA	2.8 (2.0)^a^	1.7 (1.7)	1.8 (1.7)^b^	1.8 (1.8)^c^
Muscle strengthening PA	2.3 (2.3)^a^	1.7 (2.3)	1.7 (2.3)^b^	1.5 (2.2)^c^

Sleeping/resting enough, mean days per week (SD)	3.3 (2.1)^a^	2.7 (2.2)^e^	2.9 (2.2)^b^	2.1 (1.9)^c^^,^^f^

ATOD (%)				
Never smoked, last 3 months	82.8^a^	78.4^e^^,^^h^	72.9^b^^,^^f^	49.9^c^
Never use/d drug/s	71.6^a^	77.2^e^	74.7^i^	52.8^c^^,^^f^
Moderate consumption of alcohol (*Never, less than once a week, once a week*)	61.7^a^	69.4^d^^,^^e^	58.8	33.5^c^^,^^f^
≥5 alcoholic drinks in 1 sitting, last 2 weeks	7.8^a^	0.2^d^^,^^e^	10.5	39.3^c^^,^^f^
No problem drinking (CAGE score <2)	76.8^a^^,^^j^	83.2^e^	79.3^l^	72.5^k^

*Chi square test (for categorical variables), analysis of variance (ANOVA) *post hoc* tests (for continuous variables); ATOD, alcohol, tobacco, and other drug*.

*^a^*p* < 0.001 (cluster 1 vs. 2)*.

*^b^*p* < 0.001 (cluster 1 vs. 3)*.

*^c^*p* < 0.001 (cluster 1 vs. 4)*.

*^d^*p* < 0.001 (cluster 2 vs. 3)*.

*^e^*p* < 0.001 (cluster 2 vs. 4)*.

*^f^*p* < 0.001 (cluster 3 vs. 4)*.

*^g^*p* = 0.03 (cluster 2 vs. 3)*.

*^h^*p* = 0.002 (cluster 2 vs. 3)*.

*^i^*p* = 0.031 (cluster 1 vs. 3)*.

*^j^*p* = 0.03 (cluster 1 vs. 4)*.

*^k^*p* = 0.03 (cluster 3 vs. 4)*.

*^l^*p* = 0.05 (cluster 2 vs. 3)*.

*All p-values for ANOVA post hoc tests were Bonferroni-adjusted*.

In addition, we observed significant differences by sufficiency of monthly disposable income (χ^2^ = 25.1, *p* = 0.003, *Cramer’s Phi* = 0.047). Specifically, disposable monthly income was significantly higher in Cluster 1 than Cluster 4 (χ^2^ tests, *p* = 0.002) and in Cluster 1 than Cluster 2 (χ^2^ tests, *p* = 0.029).

Further, the clusters differed significantly by type of student accommodation during the academic terms (χ^2^ = 122.4, *p* < 0.001, *Cramer’s Phi* = 0.109). All differences by type of accommodation were significant (χ^2^, *p* < 0.001) except for between Clusters 1 and 3.

Finally, all the clusters differed significantly by students’ mean age (*F* = 45.9, *p* < 0.001) whereby; Cluster 4 had the youngest student sample of 22.6 (SD 6.4) years, while Cluster 1 students exhibited the highest mean age (27, SD 10.0).

### Lifestyle Characteristics of Each Cluster

Table 3 provides a summary of the BRFs characteristics of the student clusters.

#### Cluster 1 (The High Physically Active and Health Conscious)

These students had very high health awareness/consciousness, high regard for healthy eating, and the highest fruit/vegetable consumption among all the four clusters. They were also the most physically active and had more adequate sleep compared to the other clusters. Their ATOD use was lower compared to that of Clusters 3 and 4.

#### Cluster 2 (The Abstinent)

These students had the least ATOD use compared to other clusters. Only 0.2% of this cluster had been binge drunk in the previous 2 weeks, and only 16.8% screened positive for problem drinking (CAGE test). This cluster also had the second highest percentage of nonsmokers (78.4%) within the last 3 months, and highest proportion (77.2%) of life-time never drug users. The cluster comprised highly health conscious students who regarded healthy eating as highly important, and their fruit/vegetable consumption was third highest (though much lower than cluster 1). Sweets consumption was medium high, and cluster members had a medium high mean of 2.7 days of adequate sleep per week.

#### Cluster 3 (The Moderately Health Conscious)

This cluster comprised students with the highest regard for healthy eating, second highest fruit/vegetable consumption (but much lower than cluster 1), and their consumption of sweets was lowest among all the clusters. Their ATOD use was lower than that of Cluster 4 and Cluster 1, and on average, slept adequately for 2.9 days per week. Their PA level was not particularly high and similar to that of Clusters 2 and 4 students.

#### Cluster 4 (The Risk Taking)

This cluster included students with the highest ATOD use among the 4 clusters. Likewise, these students were the least health conscious, attached the least importance to eating healthy, had the least daily fruit intake and highest intake of sweets. Although Cluster 4 members slept adequately for the least number of days (2.1), however, their PA level was similar to that of Clusters 2 and 3.

Chi square and ANOVA results (Table 3) indicated statistically significant differences between the clusters based on students’ lifestyle BRFs, with the majority of the differences across the clusters being highly statistically significant, i.e., *p* < 0.001.

### Association Between Cluster Type and Self-Rated Health

Students self-rated their health as good (41.8%), very good (38.5%), excellent (9%), fair (9.2%), or poor (1.4%). Ordinal regression examined the association between students’ self-rated health and cluster type. We found an interaction effect (χ*^2^* = 247.7, *p* < 0.001) between gender and the main exposure variable (cluster type). Therefore, we stratified the analysis by gender (Table 4). For both genders, the odds for higher self-rated health level were lower for Cluster 2, Cluster 3, and Cluster 4 compared to Cluster 1. All associations were highly statistically significant (*p* < 0.001).

**Table 4:** Associations between cluster type and students’ self-rated health among women and men.

	Self-rated health^a^			
	Women (*n* = 2,883)		Men (*n* = 823)	
Cluster type	Odds ratio (95% CI)	*p*	Odds ratio (95% CI)	*p*
1. The physically active and health conscious	Reference		Reference	
2. The abstinent	0.46 (0.38, 0.55)	<0.001	0.34 (0.24, 0.48)	<0.001
3. The moderately health conscious	0.53 (0.43, 0.66)	<0.001	0.28 (0.19, 0.42)	<0.001
4. The risk taking	0.32 (0.26, 0.39)	<0.001	0.18 (0.13, 0.26)	<0.001

*Ordinal regression models; CI: confidence interval*.

*^a^Self-rated health: ordinal dependent variable with increasing levels (poor, fair, good, very good, excellent)*.

### Association Between Cluster Type and Self-Rated Academic Performance

Participants rated their academic performance compared to their peers as “the same” (65.5%), “worse” (16.9%), “much better” (1.5%), “better” (14.1%), or “much worse” (1.9%). We found an interaction effect (χ*^2^* = 93.47, *p* < 0.001) between gender and the main exposure variable (cluster type). Therefore, we stratified the analysis by gender (Table 5). Ordinal regression showed (Table 5) that compared to Cluster 1 as reference, Clusters 2 and 4 were associated with lower odds of higher self-rated academic performance among female students. Among the male students, compared to Cluster 1 as reference, Clusters 3 and 4 were both associated with lower odds for higher self-rated academic performance.

**Table 5:** Associations between cluster type and academic performance among women and men.

	Self-rated academic performance^a^			
	Women (*n* = 2,883)		Men (*n* = 823)	
Cluster type	Odds ratio (95% CI)	*p*	Odds ratio (95% CI)	*p*
1. The physically active and health conscious	Reference		Reference	
2. The abstinent	0.79 (0.65, 0.96)	0.018	0.70 (0.49, 1.02)	0.062
3. The moderately health conscious	0.81 (0.63, 1.03)	0.091	0.66 (0.44, 0.99)	0.046
4. The risk taking	0.70 (0.56, 0.87)	0.002	0.57 (0.40, 0.81)	0.002

*Ordinal regression model; CI: confidence interval*.

*^a^Self-rated academic performance: ordinal outcome with increasing levels (much worse, worse, the same, better, much better)*.

## Discussion

We identified and described the clustering of five major health lifestyle BRFs, that included the “big four” modifiable health behaviors (ATOD, nutrition, PA, and sleep); and examined the associations of the resulting clusters with students’ self-rated health and academic performance. Multiple BRFs were prevalent in a significant proportion of our undergraduates, denoting the coexistence of health damaging lifestyle characteristics.

Our main findings include: (a) less healthy cluster membership was associated with lower self-rated health; (b) less healthy cluster membership was associated with lower academic performance; and (c) there were subgroups of students with particularly high risk for a certain BRF and for a combination of multiple BRFs. Specifically, our findings indicated that student groups with a clustering of BRFs such as “the risk taking” or “the abstinent” and even “the moderately health conscious” exhibited lower self-rated health than “the physically active and health conscious” cluster. In addition, students in these clusters reported lower academic performance, although some effects were found only for male or for female students. This implied that certain clustering of BRFs does not only diminish the health of students, but may also affect their academic achievement negatively. Young students, financially deprived students and those living with roommates or not alone were most likely to belong to these groups with higher risk profile. Therefore, universities need to be aware that large proportions of their student population may practice harmful lifestyle behaviors. Accordingly, universities need to develop targeted interventions, e.g., programs that specifically address ATOD which tends to be high in combination with poor eating habits and poor sleep among certain groups of younger students.

The current survey found distinct lifestyle behavior patterns among undergraduates. Due to different methodologies, sample selection, different BRFs examined, and the scarcity of studies that explored BRFs clustering among university students, we could compare our results only to a certain extent with other research. Generally, our findings are in line with other studies. For instance, our results are supported by research among young people (15–21 years and older) in Switzerland, where multiple risk factors were observed in a substantial proportion of young people (12). Likewise, our four clusters of BRFs resonate with an Irish study among the general population, where research of health behavior clustering in a nationally representative adult sample observed six clusters (20). Likewise, in China, a two-step CA identified three health-related lifestyle clusters (21).

In terms of university students, other research also supported our cluster findings. For instance, among young female students in the USA, where the majority of women had more than two unhealthy behaviors and a CA defined three distinct clusters (22); or among undergraduates at a university in the UK, which found three distinct student clusters based upon PA, fruit/vegetable intake, binge drinking, and smoking (4).

There is a dearth of studies on the mechanisms explaining clustering of multiple risk behaviors, even though these behaviors are significant public health issues. Some authors observed “transfer” effects (e.g., nonsmokers consume less alcohol, regularly active people smoke less, nondrinkers smoke less); as well as “compensation” effects (e.g., regularly active people consume alcohol more frequently; alcohol drinkers are more active) (23). Other research, albeit applied to one risk behavior (smoking) rather than to multiple BRFs, has highlighted the role of psychosocial and behavioral protection and risk factors (24) and has shown that such factors, theoretically derived from the constructs in problem-behavior theory (25), were associated with the initiation of smoking in a longitudinal study (24). We suggest that a framework of protective and risk factors might be helpful in exploring college multiple BRFs. Risk factors that stimulate several risk behaviors simultaneously could be social pressures to use ATOD, to consume unhealthy food and to neglect sleep. More generic protective factors, as derived from problem-behavior theory, could be values and expectations for academic achievement or support and control from parents, friends, or partners (25).

This study has some limitations. It is cross-sectional and generalizations of the findings need caution. Self-reported data could have imprecisions (recall bias, social desirability, and sociability). Students were recruited during lessons/lectures, hence those not present in the class at the time of data collection, were not included in the survey. Some variables were measured by single items due to respondent burden, and that the study was a general student health survey undertaken within a short duration during lectures. This rendered the measurement of BRFs with more items unworkable. We did not assess serving sizes in the questions on nutrition. The extent of observed clustering of more or less favorable variables might be subject to many features (usually not measured) that would confound such complex and intricately associated constellations of BRFs relationships. Such confounders are usually challenging to unpack, let alone attribute to certain aspects of the university, region, country, or participating individuals (7). Future research should attempt to address these limitations. Nevertheless, the current research is a pioneer in examining a wider range of key BRFs across a large sample of undergraduates in three UK countries; and, it is one of the few studies that systematically considered self-rated health and academic achievement of the participants.

We conclude that the identification of subgroups of young people with a high prevalence of one or more of these risk factors allows for an optimization of the allocation of preventive measures. The identification of distinct clusters may aid in uncovering specific groups with higher risk behavior profiles. The clustering of risk factors provides support for multiple-behavior interventions. Our study calls for drug prevention campaigns and other preventive activities targeted at certain sub-groups of students who are likely to combine PA with alcohol and other drug use.

## Ethics Statement

This study was carried out in accordance with the recommendations of the ethics committee of the University of Gloucestershire with informed consent from all subjects. All subjects gave informed consent in accordance with the Declaration of Helsinki. The protocol was approved by the ethics committees of the participating institutions, namely the University of Chester University of Gloucestershire, Bath Spa University, University of Ulster, Swansea University, Oxford Brookes University, and Plymouth University.

## Author Contributions

WA has conceptualized the study and is responsible for the data acquisition, interpretation of results, and drafted the manuscript. DS is responsible for the data analysis and contributed to the drafting of the manuscript. CS participated in the conceptualization of the study, the interpretation of results, and drafting of the manuscript. All authors have approved the final version of the manuscript.

## Conflict of Interest Statement

The authors declare that the research was conducted in the absence of any commercial or financial relationships that could be construed as a potential conflict of interest.

## References

[1] Stock C Mikolajczyk R Bloomfield K Maxwell AE Ozcebe H Petkeviciene J Alcohol consumption and attitudes towards banning alcohol sales on campus among European university students. Public Health (2009) 123(2):122–9. 10.1016/j.puhe.2008.12.009 19185890

[2] Wicki M Kuntsche E Gmel G. Drinking at European universities? A review of students’ alcohol use. Addict Behav (2010) 35(11):913–24. 10.1016/j.addbeh.2010.06.015 20624671

[3] El Ansari W Stock C John J Deeny P Phillips C Snelgrove S Health promoting behaviours and lifestyle characteristics of students at seven universities in the UK. Cent Eur J Public Health (2011) 19(4):197–204. 22432394 10.21101/cejph.a3684

[4] Dodd LJ Al-Nakeeb Y Nevill A Forshaw MJ. Lifestyle risk factors of students: a cluster analytical approach. Prev Med (2010) 51(1):73–7. 10.1016/j.ypmed.2010.04.005 20385163

[5] Ssewanyana D Sebena R Petkeviciene J Lukács A Miovsky M Stock C. Condom use in the context of romantic relationships: a study among university students from 12 universities in four Central and Eastern European countries. Eur J Contracept Reprod Health Care (2015) 20(5):350–60. 10.3109/13625187.2014.1001024 25664484

[6] Keller S Maddock JE Hannöver W Thyrian JR Basler H-D. Multiple health risk behaviors in German first year university students. Prev Med (2008) 46(3):189–95. 10.1016/j.ypmed.2007.09.008 18242666

[7] El Ansari W Stock C UK Student Health Group Snelgrove S Hu X Parke S Feeling healthy? A survey of physical and psychological wellbeing of students from seven universities in the UK. Int J Environ Res Public Health (2011) 8(5):1308–23. 10.3390/ijerph8051308 21655121 PMC3108111

[8] Miller NS Gold MS. Comorbid cigarette and alcohol addiction: epidemiology and treatment. J Addict Dis (1998) 17(1):55–66. 10.1300/J069v17n01_06 9549603

[9] Falk DE Yi H Hiller-Sturmhofel S An epidemiologic analysis of co-occurring alcohol and tobacco use and disorders. Alcohol Res Health (2006) 29(3):162–71. 17373404 PMC6527037

[10] Tan PN Steinbach M Kumar V Data Mining Cluster Analysis: Basic Concepts and Algorithms. New Jersey: Pearson Education Inc. (2014).

[11] Kaufman L Rousseeuw PJ Finding Groups in Data: An Introduction to Cluster Analysis. New Jersey: John Wiley & Sons (2009).

[12] Haug S Schaub MP Gross CS John U Meyer C. Predictors of hazardous drinking, tobacco smoking and physical inactivity in vocational school students. BMC Public Health (2013) 13(1):475. 10.1186/1471-2458-13-475 23672294 PMC3658930

[13] El Ansari W Stock C. Is the health and wellbeing of university students associated with their academic performance? Cross sectional findings from the United Kingdom. Int J Environ Res Public Health (2010) 7(2):509–27. 10.3390/ijerph7020509 20616988 PMC2872284

[14] El Ansari W Maxwell A Mikolajczyk R Stock C Nydenova V Krämer A Promoting student health: benefits and challenges of a Europeanwide research consortium. Cent Eur J Public Health (2007) 15(2):58–65. 17645218 10.21101/cejph.a3418

[15] El Ansari W Vodder Clausen S Mabhala A Stock C. How do I look? Body image perceptions among university students from England and Denmark. Int J Environ Res Public Health (2010) 7(2):583–95. 10.3390/ijerph7020583 20616992 PMC2872285

[16] Sarstedt M Mooi E Cluster analysis. In: Sarstedt M Mooi E, editors. A Concise Guide to Market Research. New York: Springer (2014). p. 273–324.

[17] McHugh ML The chi-square test of independence. Biochem Med (2013) 23(2):143–9. 10.11613/BM.2013.018 PMC3900058 23894860

[18] Bland JM Altman DG Multiple significance tests: the Bonferroni method. BMJ (1995) 310(6973):170 10.1136/bmj.310.6973.170 7833759 PMC2548561

[19] Graham JW Olchowski AE Gilreath TD. How many imputations are really needed? Some practical clarifications of multiple imputation theory. Prev Sci (2007) 8(3):206–13. 10.1007/s11121-007-0070-9 17549635

[20] Conry MC Morgan K Curry P McGee H Harrington J Ward M The clustering of health behaviours in Ireland and their relationship with mental health, self-rated health and quality of life. BMC Public Health (2011) 11(1):692. 10.1186/1471-2458-11-692 21896196 PMC3187756

[21] Lv J Liu Q Ren Y Gong T Wang S Li L Socio-demographic association of multiple modifiable lifestyle risk factors and their clustering in a representative urban population of adults: a cross-sectional study in Hangzhou, China. Int J Behav Nutr Phys Act (2011) 8(1):40. 10.1186/1479-5868-8-40 21569614 PMC3117756

[22] Quintiliani L Allen J Marino M Kelly-Weeder S Li Y. Multiple health behavior clusters among female college students. Patient Educ Couns (2010) 79(1):134–7. 10.1016/j.pec.2009.08.007 19767168 PMC5892440

[23] Nigg CR Lee H-R Hubbard AE Min-Sun K. Gateway health behaviors in college students: investigating transfer and compensation effects. J Am Coll Health (2009) 58(1):39–44. 10.3200/JACH.58.1.39-44 19592352

[24] Costa FM Jessor R Turbin MS. College student involvement in cigarette smoking: the role of psychosocial and behavioral protection and risk. Nicotine Tob Res (2007) 9(2):213–24. 10.1080/14622200601078558 17365752

[25] Jessor R Problem-behavior theory, psychosocial development, and adolescent problem drinking. Br J Addict (1987) 82(4):331–42. 10.1111/j.1360-0443.1987.tb01490.x 3472582

